# 46, XX Ovotesticular disorder of sex development (true hermaphroditism) with seminoma: A case report

**DOI:** 10.1097/MD.0000000000022530

**Published:** 2020-10-02

**Authors:** Zixiang Li, Junjie Liu, Yunpeng Peng, Renfu Chen, Peng Ge, Junqi Wang

**Affiliations:** Department of Urology, the Affiliated Hospital of Xuzhou Medical University, Xuzhou, Jiangsu, China.

**Keywords:** disorder of sex development, seminoma, true hermaphroditism, testosterone replacement, case report

## Abstract

**Rationale:**

Ovotesticular disorder of sex development (DSD), previously known as true hermaphroditism, is a disorder in which individuals have both testicular and ovarian tissues. Instances of tumors arising in the gonads of individuals with 46,XX ovotesticular DSD are uncommon.

**Patient concerns:**

We report a case of a 36-year-old phenotypical male with a chief complaint of an abdominal mass for 3 months. He reported normal erections and regular menses. Computerized tomography showed a large tumor measuring 15 × 10 cm in size, a uterus, and a cystic ovary.

**Diagnosis:**

46, XX ovotesticular DSD with seminoma.

**Interventions:**

The patient was treated with neochemotherapy (etoposide and cisplatin), surgery, chemotherapy, and testosterone replacement.

**Outcomes:**

At the 13-month follow-up, the patient reported satisfactory erections, and no evidence of disease was found.

**Conclusion:**

Cases of 46,XX ovotesticular DSD with seminoma are uncommon. Our case reveals the importance of surgery combined with neochemotherapy, chemotherapy, and testosterone replacement in these patients to improve the prognosis.

## 1 Introduction

Disorder of sex development (DSD) is an umbrella term that encompasses a broad spectrum of conditions in which chromosomal, gonadal, or phenotypic (genital) sex are atypical.^[1]^ Disorders of sex development (DSDs) are classified into 3 categories, namely, sex chromosome DSD, 46,XY DSD, and 46,XX DSD.^[2]^ Ovotesticular DSD (OT-DSD), previously known as true hermaphroditism, describes individuals who have both testicular and ovarian tissues, with an overall incidence of 1 out of 100,000 live births.^[3]^

It is generally thought that testis-specific protein Y-linked region (TSPY) increases the risk of gonadal germ cell tumor (GCT) in patients with DSDs. Thus, 46, XX DSD patients who are not mosaic for TSPY are not at a higher risk of cancer.^[4]^ Instances of tumors arising in the gonads of individuals with 46,XX OT-DSD are uncommon.[^5^,^6^,^7^,^8^,^9^,^10^] Herein, we present a case of a a male phenotype 46, XX OT-DSD suffering from seminoma, who was successfully treated with surgery combined with neochemotherapy, chemotherapy, and testosterone replacement. The patient has provided informed consent for this study.

## 2 Case report

**Figure 1:**
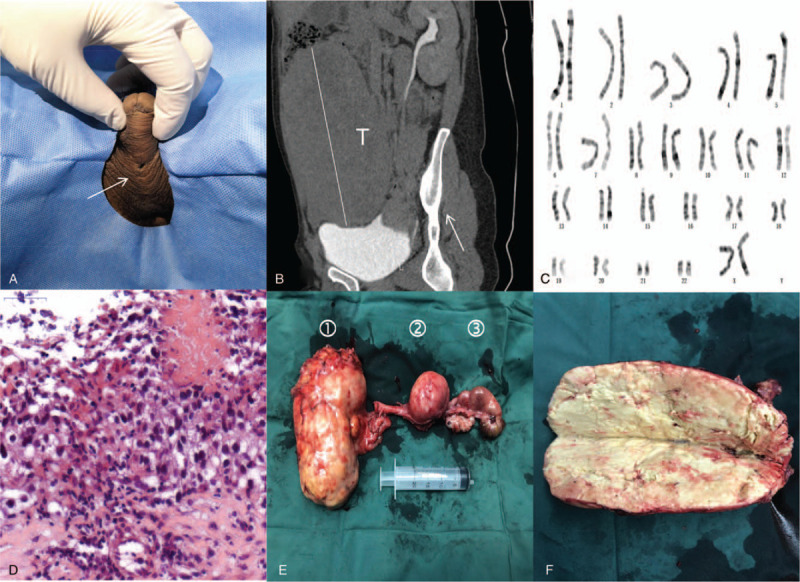
Clinicopathological features. A. Physical examination reveals a urethral fistula on the ventral surface (arrow). B. Computerized tomography shows a large tumor (T, B). C. Karyotype analysis of the patient. D. Photomicrograph of biopsied tissue shows seminoma. E. Image shows the tumor (①), uterus (②), and tubo-ovarian nodule (③). F. Image shows specimen bisection and extensive necrosis.

A 36-year-old phenotypical male who presented with a chief complaint of an abdominal mass for 3 months was admitted to the Affiliated Hospital of Xuzhou Medical University on 30 August 2018. The patient was married and had no biological children. He reported normal erections. There was no remarkable history in the family. His height was 170 cm, and his weight was 51 kg. Physical examination revealed a firm mass in the right abdomen, which was slightly movable. A surgical scar and a urethral fistula on the ventral surface **(** Fig. 1A) were found, and testes were absent. He reported urine leakage and regular menses through the fistula. His past medical history indicated that he was diagnosed with “hypospadia and bilateral cryptorchidism” when he was 12 years old and underwent hypospadias urethroplasty (details unavailable). The appearance of the external genitalia was phenotypical male, and the penis was well developed. The urethral orifice was in the normal position. The secondary sexual characteristics were normal, and no gynecomastia was found.

**Figure 2:**
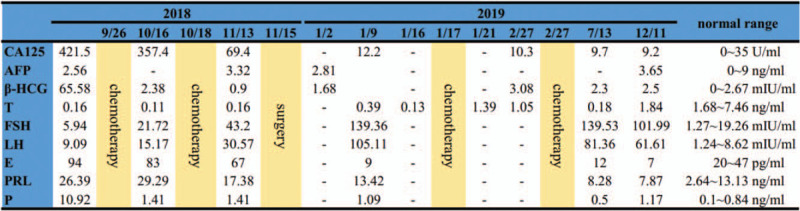
Changes in the levels of hormonal and tumor markers at 13-month follow-up. AFP = α-fetoprotein, β-HCG = β-human chorionic gonadotropin, CA125 = carbohydrate antigen 125, E = estradiol, FSH = follicle stimulating hormone, LH = luteinizing hormone, PRL = prolactin, T = testosterone.

Abdominopelvic computerized tomography showed a large tumor measuring 15 × 10 cm and occupying the abdominopelvic cavity (Fig. 1B), a uterus, and a cystic ovary on the left side. Chest computerized tomography showed no evidence of metastasis. Biochemical assessment showed decreased testosterone (0.16 ng/mL) with increased prolactin (26.39 ng/mL), estradiol (94 pg/mL), progesterone (10.92 ng/mL), β-human chorionic gonadotropin (β-HCG, 65.58 mIU/mL), and carbohydrate antigen 125 (CA125, 421.5 U/mL). Other markers, such as α-fetoprotein (AFP) and follicle stimulating hormone, were within normal ranges **(** Fig. 2). Chromosome analysis revealed a 46, XX karyotype (Fig. 1C). No Y chromosome was found. The results of the polymerase chain reaction amplification of peripheral leukocyte DNA with primers specific for sex determining region of the Y chromosome (SRY) were negative.^[11]^

In view of these findings and the probability of malignancy, the patient underwent ultrasound-guided biopsy. A pathological examination demonstrated seminoma **(** Fig. 1D). The immunohistochemistry results were as follows: Sal-like protein 4 (SALL 4, +), placental alkaline phosphatase (PLAP, +), vimentin (partially +), antigen Ki67 (Ki67, +, approximately 40%), cluster of differentiation (CD) 30 (−), CD117 (+), CD20 (−), CD3 (−), CD5 (−), CD56 (−), AFP (−), cytokeratin (CK) 8/18 (−), CK7 (−), CK20 (−), CK high molecular weight (CKH, −), CK low molecular weight (CKL, −), CK pan (−), leukocyte common antigen (LCA, −), Sox-10 (−), synaptophysin (SYN, −), GATA3 (−), soluble protein-100 (S-100, −), human melanoma black 45 (HMB45, −), anaplastic lymphoma kinase (ALK, −), and paired-box 8 (PAX8, −). The clinical stage of the patient was T1N0M0.

The patient received 2 cycles of etoposide and cisplatin (etoposide: 100 mg, day 1-day 5; cisplatin: 100 mg, day 1) before surgery. Tumor response to chemotherapy was evaluated according to the Response Evaluation Criteria in Solid Tumors (RECIST version 1.1). After 2 cycles of chemotherapy, the tumor size decreased to 9 × 6 cm. CA125 and β-HCG levels also decreased **(** Fig. 2). Open cryptorchidectomy (tumor), hysterovaginectomy, and salpingo-oophorectomy were successfully performed on 13 November 2018 **(** Fig. 1E, F). No evidence of metastasis was found. A pathological examination revealed the presence of a small uterus, fallopian tubes, and a cystic ovary. The mass was comprised of necrotic and scar tissue without residual seminoma. The patient was discharged after surgery with no perioperative complications.

The patient received 2 additional cycles of chemotherapy at 2 months after surgery, and there was no evidence of disease at 13-month follow-up. Tumor markers, such as AFP, β-HCG, and CA125, were all normal. The patient was prescribed testosterone to maintain general health. He reported satisfactory erections.

## 3 Discussion

In OT-DSD, approximately 60% of patients have 46, XX, 33% have mosaic or chimeric karyotypes carrying a Y chromosome (45,X/46,XY, 46,XX/47,XXY), and only 7% have 46,XY. Previous studies have reported SRY as the testis-determining factor. However, most individuals with OT-DSD have no separate Y chromosome, and the translocation of SRY to the X chromosome or an autosome is a common mechanism. Recent evidence indicated that SRY is permissive but not mandatory for male development.^[6]^ Other causes of XX OT-DSD include mutations in *NR5A1*, aberrant expression of *SOX3*, duplication of *SOX9*, and mutations in *RSPO1* and *WNT4*.[^12^,^13^]

Gender assignment is the most important aspect in the management of OT-DSDs. However, there is no uniform consensus regarding the assignment and surgery.^[2]^ In Africa, due to cultural factors, most 46,XX OT-DSDs are reared as males; however, in countries beyond Africa, these individuals tend to be reared as females.^[12]^ Ideally, a multidisciplinary team consisting of specialists in urology, gynecology, endocrinology, psychology, psychiatry, radiology, pathology, genetics, and clinical biochemistry should be mandatory for the assessment and care of such patients.^[2]^ Most importantly, the functional potential of the internal and external genitalia should be determined,^[14]^ as fertility is often impaired in cases of OT-DSD for both males and females. Although females can be fertile, that is, if the internal genitalia are appropriate,^[14]^ an intact Y chromosome is required for spermatogenesis. Therefore, 46,XX OT-DSD causes male infertility. Our patient was raised as a male and had no biological children.

Current knowledge indicates that patients with DSDs are at an increased risk for the development of malignancy such as seminoma, gonadoblastoma, and dysgerminoma.^[15]^ The risk for tumor development depends on many parameters, including gonadal morphology, germ cell location, presence of germ cells, genital morphology, patient age, and presence of TSPY.[^4^,^16^] Furthermore, the risk for GCT development varies among different DSDs. Cools and colleagues divided DSDs into several risk groups for the development of type II GCT^[4]^ and reported that OT-DSD patients were at a low risk of GCT development. The malignancy risk is 3% in 46,XY OT-DSD and even lower in 46,XX OT-DSD. To date, less than ten cases of tumors arising in the gonads of individuals with 46,XX OT-DSD have been reported.[^5^,^6^,^7^,^8^,^9^,^10^] As shown in Table 1, all adults were classified as male. Five out of 7 patients presented with seminoma. The median age was 35 years (range, 2 months–56 years). Surgery was the preferred option, followed by surgery with chemotherapy.

**Table 1:**
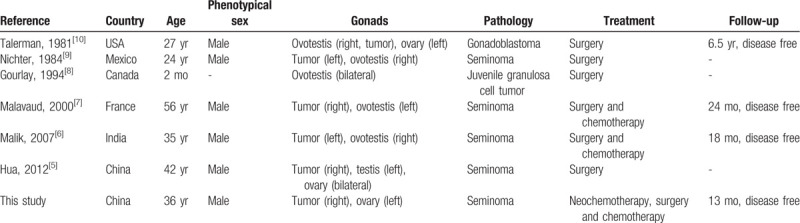
Main findings of published studies on tumors arising in the gonads of individuals with 46, XX true hermaphroditism.

Cryptorchidism, a common congenital abnormality, accounts for 9.7% of 46,XX DSD males.^[17]^ Regardless of the DSD diagnosis, the relative risk of cancer in cases of undescended testes is estimated to range from 2.75 to 8.^[15]^ A previous study has indicated that seminomas detected in individuals with cryptorchidism tended to be relatively advanced with evidence of metastasis.^[7]^ For patients with life-threatening metastases, delayed orchiectomy after prior chemotherapy (neoadjuvant chemotherapy) has been attempted. Reddy et al. reported a high complete response rate of 81.3% in patients with advanced seminoma after neoadjuvant chemotherapy.^[18]^ In our patient, there was no evidence of metastasis, and after considering the ectopic tumor location and size, neoadjuvant chemotherapy with etoposide and cisplatin was administered. After two cycles of neoadjuvant chemotherapy, the tumor size was significantly reduced from 15 × 10 cm to 9 × 6 cm. Postoperative findings of specimens showed a complete response, and only necrotic and scar tissue was found. In stage I seminoma, adjuvant chemotherapy and radiotherapy could reduce the recurrence risk by 80%.^[19]^ Therefore, 2 additional cycles of chemotherapy were administered. To the best of our knowledge, this is the first case of a phenotypical male suffering from 46, XX OT-DSD and seminoma, who was treated with surgery combined with neochemotherapy and chemotherapy.

Hormonal replacement therapy is needed for the majority of DSD conditions. Instances of seminoma in patients with 46,XX OT-DSDs are rare. For these patients, lifelong hormonal replacement therapy is often indicated after surgery. Our patient was prescribed testosterone to maintain general health; however, he took medications irregularly, partly because of forgetting to take them. At the 13-month follow-up period, the patient reported satisfactory erections, and no evidence of disease was found.

## 4 Conclusions

Cases of 46, XX ovotesticular DSD with seminoma are uncommon. Our case reveals the importance of surgery combined with neochemotherapy, chemotherapy, and testosterone replacement in these patients to improve the prognosis. Further studies are needed to guide clinicians in the appropriate management of these patients.

## Author contributions

**Conceptualization:** Peng Ge, Junqi Wang, Zixiang Li, Junjie Liu.

**Data curation:** Zixiang Li, Peng Ge.

**Resources:** Junjie Liu, Yunpeng Peng.

**Supervision:** Peng Ge, Renfu Chen, Junqi Wang.

**Writing – original draft:** Zixiang Li.

**Writing – review & editing:** Peng Ge.
